# RACE AND DETERMINANTS OF HEALTH MODERATE ASSOCIATIONS BETWEEN COGNITIVE DECLINE AND DRIVING MOBILITY OVER 10 YEARS

**DOI:** 10.1093/geroni/igac059.2951

**Published:** 2022-12-20

**Authors:** Katie Wheeler, Caitlin Pope, Tyler Bell, Brooke Carroll, Lesley Ross, Karlene Ball

**Affiliations:** University of Alabama at Birmingham, Birmingham, Alabama, United States; University of Kentucky, Lexington, Kentucky, United States; University of California, San Diego, La Jolla, California, United States; University of Alabama at Birmingham, Birmingham, Alabama, United States; Clemson University, Clemson, South Carolina, United States; University of Alabama at Birmingham, Birmingham, Alabama, United States

## Abstract

The ability to drive a vehicle is an everyday function that helps older adults maintain independence. Few observational studies have examined the relationship between cognitive decline and driving mobility and in context of racial differences and social determinants of health (SDOH). To address this empirical gap, this study aimed to characterize how cognitive functioning is longitudinally associated with driving mobility (driving space, driving exposure, and driving difficulty) in older age, and how it may vary by race and SDOH. Using the control arm of the Advanced Cognitive Training in Vital Elderly study (n=581, 24.5% Black), multilevel models examined longitudinal associations between processing speed, visual attention, memory, and reasoning with driving mobility outcomes. Race and SDOH moderations were explored. Only declines in reasoning and processing speed related to driving mobility, moderated by race and SDOH. Reasoning decline related to increased driving space in White (β=-.21,p=.006) but not Black older adults (p=.286). Processing speed decline related to greater driving exposure in Black older adults (β=-.15,p < .001) but less driving exposure in White older adults (β=.13,p=.006). Processing speed decline related to reduced driving exposure (β=-.06,p=.001) and increased driving difficulty (β=-.35,p < .001), but only in people living in poorer neighborhood and built environment and poorer social community contexts, respectively. Overall, findings emphasize that relationships between cognitive decline and driving mobility are dependent on race and SDOH. Consideration of such factors may help target those in greatest need to sustain safe driving mobility and functional independence.

